# Full-Field Assessment of Damage Evolution in Compressed Masonry with Bed Joint Reinforcement Using Digital Image Correlation

**DOI:** 10.3390/ma19061145

**Published:** 2026-03-15

**Authors:** Artur Piekarczuk, Przemysław Więch, Jacek Głodkiewicz

**Affiliations:** Building Research Institute, 00-611 Warsaw, Poland

**Keywords:** digital image correlation, damage evolution, material health monitoring, masonry, autoclaved aerated concrete, crack morphology, strain localisation, bed joint reinforcement, compressive loading

## Abstract

**Highlights:**

- Bed joint reinforcement does not increase the compressive strength of masonry.
- Steel truss reinforcement promotes the redistribution of compressive strains.
- DIC reveals strain localisation patterns not visible in global measurements.
- Bed joint reinforcement acts mainly as crack control rather than strengthening.
- Full-field DIC improves assessment of damage evolution in masonry.
- Global force-based indicators may not capture damage mechanisms.

**Abstract:**

This experimental study investigates the influence of selected bed joint reinforcement systems on the evolution of damage and crack development in masonry elements subjected to axial compression. Autoclaved aerated concrete masonry samples reinforced with steel truss reinforcement, unidirectional carbon fibre mesh and steel cords embedded in a fibreglass matrix were tested and compared to an unreinforced reference specimen. Full-field deformation and strain localisation were monitored using digital image correlation (DIC). The results indicate that bed joint reinforcement does not lead to a measurable increase in compressive load-bearing capacity, as differences in ultimate load remain within experimental uncertainty. However, clear differences in the evolution and spatial distribution of damage were observed. Steel truss reinforcement promoted strain redistribution and delayed localisation of tensile strains, while the remaining reinforcement systems exhibited only limited influence on crack morphology. The findings confirm that bed joint reinforcement in compressed masonry should be classified as a nonstructural solution and demonstrate the diagnostic value of full-field deformation monitoring for assessing damage evolution and crack control in masonry structures.

## 1. Introduction

Masonry structures constitute one of the oldest construction systems in civil engineering and remain widely used in residential, building, and infrastructural applications due to favourable material characteristics, including durability, fire resistance, availability of raw materials, and satisfactory thermal insulation performance [1]. The contemporary development of masonry technologies is increasingly based on the introduction of new materials and construction solutions, whose effectiveness is verified through experimental and analytical research [2]. Despite continuous technological progress, masonry remains susceptible to degradation processes induced by environmental exposure, service conditions, and sustained loading. These degradation mechanisms manifest themselves primarily through the initiation and propagation of cracking, which governs the serviceability limit state and, under unfavourable conditions, may ultimately lead to a loss of structural safety.

From a material behaviour perspective, analysis of the initiation, propagation, and location of damage in masonry elements is of fundamental importance, particularly under compression-dominated loading. Conventional global response measures, such as ultimate load capacity or secant elastic modulus, do not provide sufficient information on the early stages of material degradation or the mechanisms that govern damage redistribution. Consequently, it is necessary to distinguish between reinforcement solutions of a structural nature, which directly influence load-bearing capacity, and nonstructural solutions, whose effect is limited to modifying the degradation process and controlling crack development. This distinction is especially relevant for masonry elements subjected predominantly to compressive loading, where a strengthening effect is often difficult to demonstrate using force-based criteria alone.

The literature reports a wide range of masonry reinforcement and reinforcement techniques. Invasive methods are based on internal reinforcement systems [3,4], while non-invasive approaches rely on externally applied strengthening layers in the form of reinforced mortars or plasters with steel meshes [5], natural fibres [6] polypropylene bands [7,8], carbon-fibre-reinforced polymers (CFRPs) [9], glass-fibre-reinforced polymers (GFRPs) [10] and high-performance polymer fibres such as ZylonTM [11,12]. Hybrid solutions that combine surface reinforcement with bed joint reinforcement have also been proposed [13]. Studies conducted by researchers from the Silesian University of Technology [14] demonstrated that both the type of horizontal joint and the applied bed joint reinforcement influence crack propagation mechanisms and stress redistribution in masonry walls. Subsequently, these findings were synthesised in a monograph on crack mitigation in compressed masonry [15].

In engineering practice, steel reinforcement remains the predominant solution, whereas alternative reinforcement materials are applied to a limited extent, particularly within horizontal joints. Harmonised European standard EN 845-3 [16] defines a clear distinction between structural reinforcement, intended to enhance load capacity, and non-structural reinforcement, whose primary function is crack control. Composite reinforcement systems may be particularly relevant within the latter category; however, their actual influence on material degradation processes and damage evolution in compression-governed masonry remains not sufficiently quantified. Parallel to the development of reinforcement solutions, significant progress has been achieved in diagnostic methods that enable the monitoring of deformation and damage both on local and global scales. Among these, Digital Image Correlation (DIC) provides non-contact full-field measurements of strain fields and enables identification of damage localisation throughout the loading history [17].

Recent studies have increasingly applied digital image correlation to investigate deformation and cracking processes in masonry elements and structural components [18,19]. Full-field measurements obtained using DIC enable detailed identification of strain localisation patterns, crack initiation zones, and their evolution during loading. Such approaches have been successfully applied in experimental investigations of masonry walls and reinforced masonry elements, providing detailed information on deformation fields and crack development mechanisms. Previous studies have demonstrated the usefulness of DIC in analysing the localisation of deformation in masonry components and in investigating the interaction between reinforcement systems and masonry substrates. These studies confirm that full-field optical measurements provide information that cannot be obtained using conventional point-based sensors, such as strain gauges or LVDTs. Despite the increasing use of DIC in masonry research, limited experimental data are available on the influence of different bed joint reinforcement systems on strain localisation and damage evolution in compression-dominated masonry elements.

The increasing availability of commercial measurement systems, such as ARAMIS, has facilitated the widespread application of DIC in experimental investigations of structural materials and structural components, including the analysis of deformation and cracking in masonry structures [20,21] as well as related masonry-based structural systems [22]. From a material health monitoring perspective, DIC allows the identification of damage initiation and the tracking of its spatial evolution, which cannot be captured using conventional point-based measurement techniques. The present study is devoted to a full field assessment of the evolution of damage in autoclaved aerated concrete masonry elements subjected to compressive loading, using DIC as a diagnostic and monitoring tool. The applied bed joint reinforcement systems are treated as parameters that influence the degradation process and damage redistribution, rather than as measures designed to increase compressive load-bearing capacity. The objective of this work is to provide a qualitative and quantitative analysis of crack morphology and strain localisation based on full-field measurements, contributing to the development of material health assessment and crack control approaches for non-structural masonry reinforcement solutions.

## 2. Materials and Methods

### 2.1. Materials and Specimen Preparation

**Figure 1:**
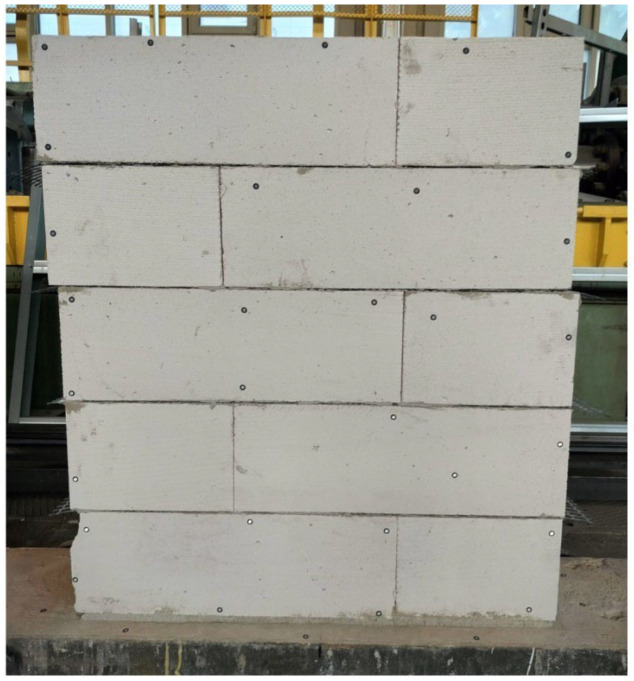
Standard model of masonry with thin bed joints.

The experimental programme comprised four masonry specimens, including one unreinforced reference specimen and three specimens that incorporate different types of bed joint reinforcement. For each configuration, three independent samples (*n* = 3) were tested. All samples were prepared according to EN 1052-1 [23] and represented wall elements with approximate dimensions of 0.9 m × 1.0 m, composed of five layers of masonry units. A standard masonry configuration with thin bed joints was adopted as the reference construction solution (Figure 1).

Autoclaved aerated concrete (AAC) blocks were used as masonry units. The selection of AAC was based on its favourable bonding behaviour with thin-layer cement mortars, as confirmed by the authors in an extensive series of previous experimental investigations. The blocks had nominal dimensions of 599 mm × 240 mm × 199 mm, a declared dry density of 400 kg/m^3^ and a nominal compressive strength of 2.5 N/mm^2^. The actual mean compressive strength of the units, determined according to EN 772-1 [24], was equal to 2.7 N/mm^2^.

**Figure 2:**
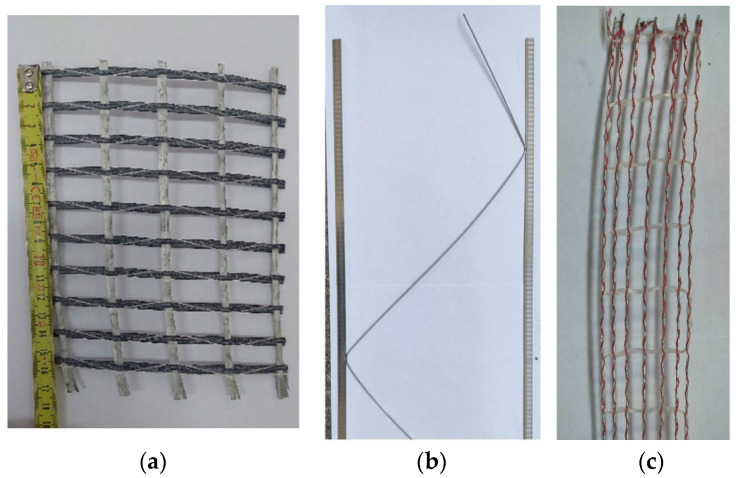
Reinforcing materials for masonry bed joints: (**a**) unidirectional carbon fibre mesh, (**b**) steel bed joint reinforcement in the form of a truss, and (**c**) mesh composed of steel cables embedded in a fibreglass matrix.

Mortar was applied exclusively to the horizontal bed joints. A factory-prepared single-component thin-layer cement mortar with a declared compressive strength of 10 N/mm^2^ was used. The actual mean compressive strength of the mortar, measured according to EN 1015-11 [25], amounted to 13.3 N/mm^2^. Three different reinforcement systems were introduced into the horizontal bed joints (Figure 2):

(a) ARMO Mesh 200/200 unidirectional carbon fibre mesh (S&P), embedded in a polymer matrix.
(b) Steel truss bed joint reinforcement Murfor^®^ EFS/Z 190 × 3050; flat strip dimensions 8 mm × 1.5 mm; minimum tensile strength 550 N/mm^2^; minimum yield strength 500 N/mm^2^; minimum weld shear resistance 2500 N.
(c) Murfor Compact I-50 consisting of steel wires embedded in a polymer coating (‘Nova-Elewacje’).

In all reinforced specimens, the reinforcement was placed within double-layer bed joints. The construction procedure consisted of applying mortar to the upper surface of the lower masonry unit, placing the reinforcement cut to the required dimensions and subsequently positioning the upper masonry unit with mortar applied to its bottom surface. This procedure resulted in a double-layer joint with a total thickness ranging from 4.0 to 4.5 mm, with the reinforcement fully embedded in mortar on both sides. An identical double-layer joint configuration, but without reinforcement, was used in the reference specimen to ensure consistency of joint geometry.

After construction, all samples were stored under laboratory conditions for a minimum curing period of 28 days. During curing, the ambient temperature ranged from 21.4 °C to 22.9 °C, while the relative humidity ranged between 44.3 and 58.5%. The samples were tested in equilibrium with laboratory ambient conditions without additional moisture conditioning prior to testing.

### 2.2. Test Setup and Measurement System

Mechanical tests were performed using a 5 MN Amsler hydraulic testing machine (Amsler, Feuerthalen, Switzerland) calibrated according to ISO 7500-1 (Class 1) [26] within a measurement range of 500 kN. The machine was equipped with upper and lower spherical bearings to minimise load eccentricities and ensure uniform stress distribution during axial compression. The samples were subjected to monotonic axial compression until failure. The load was applied under load control, acknowledging the inherent limitations of hydraulic systems related to the finite oil flow rate. The load rate was maintained at approximately 3 kN/s. The applied load was determined from hydraulic oil pressure and recorded using an HBM MGCplus data acquisition system (HBM GmbH, Darmstadt, Germany). Specimens were subjected to monotonic axial compression until failure without unloading cycles.

**Figure 3:**
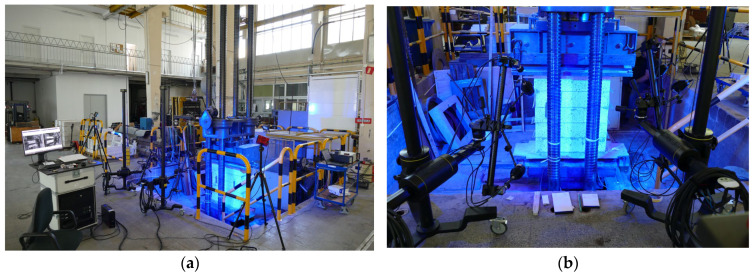
Sample during testing: (**a**) general arrangement of the test setup and (**b**) ARAMIS sensor configuration.

The general arrangement of the test setup and the positioning of the optical measurement system are shown in Figure 3a,b.

For synchronisation purposes, the load signal was converted into a conditioned voltage output and supplied directly to the digital image correlation (DIC) system, ensuring the consistent acquisition of mechanical and optical data throughout the loading history. Full-field deformation measurements were performed using the ARAMIS DIC system (GOM GmbH, Braunschweig, Germany). Two independent sensor units were employed, each consisting of a pair of cameras. The use of two sensors was required due to the partial obstruction of the specimen surface by the columns of the hydraulic press (Figure 3b). Each camera was equipped with a sensor resolution of 4096 × 3000 pixels and a lens with a focal length of 12 mm. The aperture was set to f/5.6. The camera spacing was equal to 688 mm, resulting in a working distance of approximately 1640 mm. The corresponding measurement volume was 1845 × 1490 × 1490 mm, which produced a spatial resolution of approximately 2.2 pixels/mm. A random speckle pattern was applied manually to the surface of each sample to enable optical correlation. Minor variations in the speckle quality between samples resulted from the manual application process; however, the quality of the pattern was verified prior to each test to ensure reliable correlation results. To provide independent verification of DIC-based deformation measurements, three reinforced specimens were additionally instrumented with linear variable differential transformer (LVDT) displacement transducers. The effective gauge length of the LVDTs was approximately 400 mm. LVDT measurements were used exclusively for validation purposes and were not used as primary indicators of deformation or damage evolution. The load and displacement signals acquired by the MGCplus system were processed using built-in low-pass Bessel filtering to reduce high-frequency hydraulic noise. No additional numerical smoothing was applied to the strain fields obtained from the DIC analysis beyond the standard spatial correlation procedures implemented in the ARAMIS software v 2022 (GOM GmbH, Braunschweig, Germany).

## 3. Results

The experimental results are presented in the form of load–deformation relationships, full-field strain maps obtained using digital image correlation (DIC), and a summary of key mechanical parameters. For each specimen, the results include:

(a) load–strain curves derived from virtual DIC strain gauges and, where applicable, from physical LVDT measurements;
(b) the spatial arrangement of virtual DIC strain gauges and LVDT transducers,
(c) maps of the principal tensile strain,
(d) Maps of principal compressive strain corresponding to the maximum applied load.

The principal tensile strain fields are used as indicators of the onset and development of cracking on the observed surface of the masonry specimens.

A quantitative summary of the ultimate load, ultimate stress, and secant elastic modulus values is provided in Table 1.

Crack initiation was identified on two complementary criteria. First, the deviation from the initial quasi-linear load–vertical strain relationship was interpreted as the onset of stiffness degradation. Second, the appearance of localised principal tensile strain concentrations on DIC strain maps forming continuous localisation bands was treated as an indicator of crack initiation and propagation. The combination of global mechanical response and full-field strain localisation allowed consistent identification of damage onset across all specimens.

### 3.1. Reference (Unreinforced) Specimen

**Figure 4:**
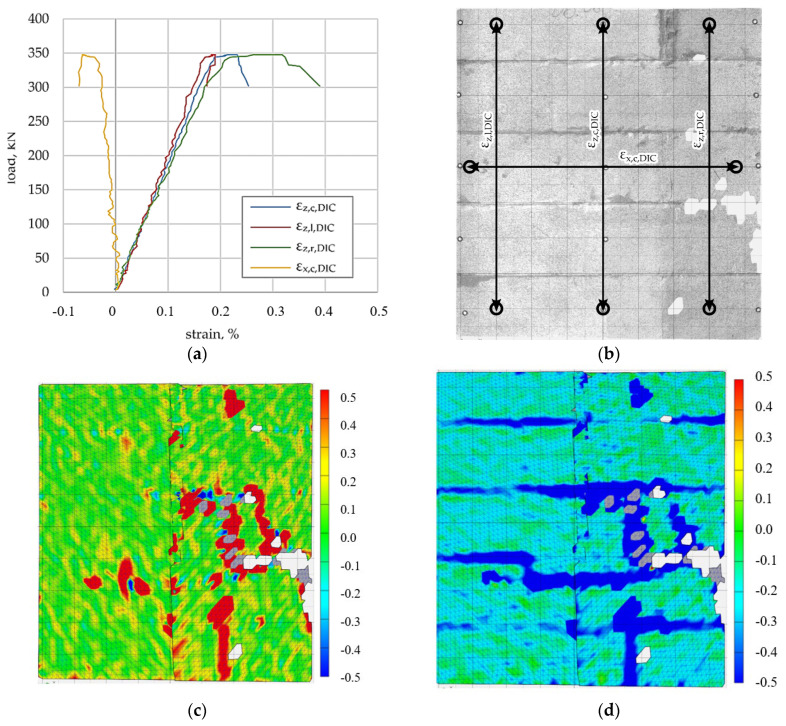
Reference specimen (unreinforced), (**a**) load–deformation relationship, (**b**) placement of DIC virtual strain gauges; arrows indicate the measurement directions of virtual gauges and circles denote the gauge endpoints., (**c**) principal tensile strain under maximum load, (**d**) principal compressive strain under maximum load.

**Figure 5:**
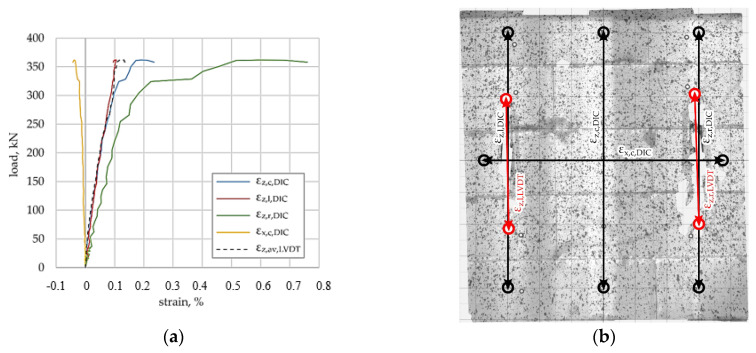

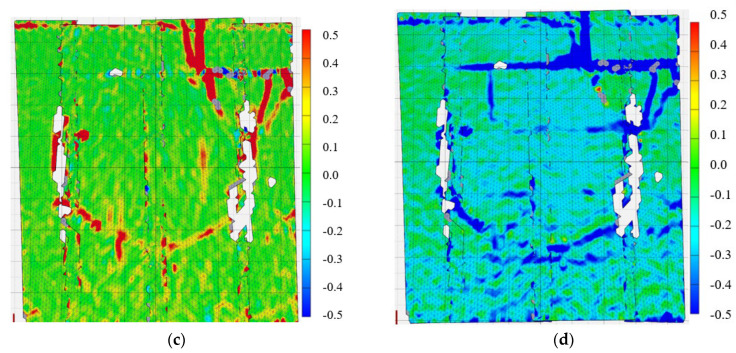
Steel-truss-reinforced specimen, (**a**) load–deformation relationship, (**b**) placement of virtual DIC virtual and LVDT strain gauges (black and red, respectively), (**c**) principal tensile strain under maximum load, (**d**) principal compressive strain under maximum load.

**Figure 6:**
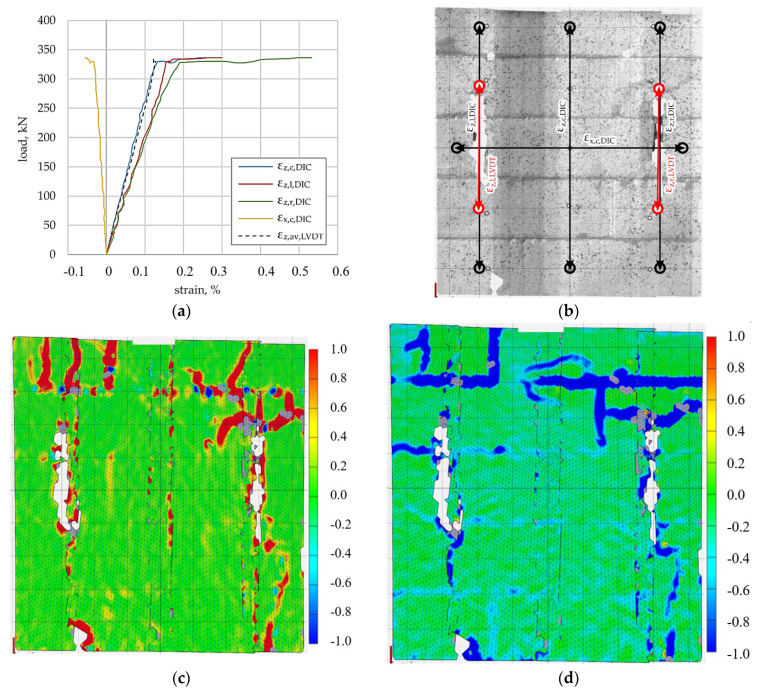
Sample reinforced with a net of steel cords with cross-glass roving, (**a**) load–deformation relationship, (**b**) placement of virtual DIC and LVDT strain gauges (black and red, respectively), (**c**) principal tensile strain under maximum load, (**d**) principal compressive strain under maximum load.

**Figure 7:**
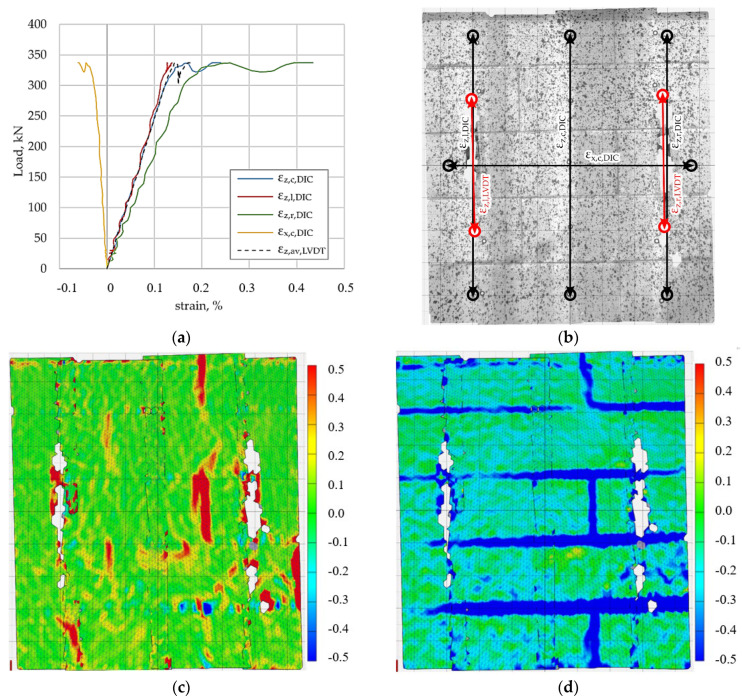
Sample reinforced with unidirectional carbon fibre mesh, (**a**) load–deformation relationship, (**b**) placement of virtual strain gauges DIC and LVDT strain gauges (black and red, respectively), (**c**) principal tensile strain under maximum load, (**d**) principal compressive strain under maximum load.

The load–deformation response of the unreinforced reference specimen is shown in Figure 4a. Vertical strain components obtained from DIC measurements exhibit a nearly linear response up to maximum load, followed by a rapid loss of stiffness associated with crack development. No LVDT measurements were available for the reference specimen. In addition to visual interpretation of the strain maps, particular attention was paid to the magnitude and spatial distribution of the principal tensile strains obtained from the DIC analysis. Zones characterised by elevated principal tensile strain values were interpreted as indicators of strain localisation and potential crack initiation. The strain maps presented in Figure 4, Figure 5, Figure 6 and Figure 7 correspond to representative results obtained from individual specimens tested for each reinforcement configuration.

The arrangement of virtual DIC strain gauges and the applied speckle pattern are shown in Figure 4b. At the maximum load level, the principal tensile strain map (Figure 4c) reveals localised zones of increased tensile strain, predominantly aligned with the horizontal bed joints and locally extending into the masonry units. These zones, characterised by elevated principal tensile strain values, correspond to the formation and coalescence of cracks on the surface of the specimen and therefore indicate regions of strain localisation and crack initiation. The principal compressive strain map (Figure 4d) shows a relatively uniform compressive strain distribution, with localised concentrations associated with the tensile strain localisation.

### 3.2. Steel Truss Reinforced Specimen

The load–deformation relationship for the specimen reinforced with steel truss bed joint reinforcement is presented in Figure 5a. In addition to the DIC-derived strains, the vertical strains measured by LVDT transducers are included for comparison. The DIC and LVDT measurements exhibit comparable trends over the analysed load range.

The locations of the virtual DIC strain gauges and the LVDT transducers are shown in Figure 5b. The principal tensile strain map at maximum load (Figure 5c) indicates a more distributed strain field compared to the reference specimen, with localisation zones developing along several bed joints rather than forming a single dominant crack path. The corresponding compressive strain map (Figure 5d) shows a relatively uniform distribution with localised concentrations associated with the tensile strain zones. These observations suggest that steel truss reinforcement contributes to a redistribution of deformation and delays the formation of a dominant localisation band.

### 3.3. Specimen Reinforced with Steel Cords in Fibreglass Matrix

Figure 6a presents the load–deformation response of the specimen reinforced with a mesh composed of steel cords embedded in a fibreglass matrix. Vertical strains derived from DIC and measured from LVDT are shown. The global response is characterised by a gradual reduction in stiffness before reaching the maximum load.

The arrangement of the virtual DIC strain gauges and LVDT transducers is shown in Figure 6b. The principal tensile strain map at maximum load (Figure 6c) reveals several localised tensile strain zones concentrated near the reinforced bed joints and extending locally into the masonry units. Compared to steel truss reinforcement, the strain field appears more concentrated, indicating a less effective redistribution of deformation. The principal compressive strain map (Figure 6d) exhibits localised compressive bands associated with the regions of tensile strain localisation.

### 3.4. Specimen Reinforced with Unidirectional Carbon Fibre Mesh

The load–deformation relationship for the specimen reinforced with unidirectional carbon fibre mesh is shown in Figure 7a. Vertical strains obtained from DIC and LVDT measurements demonstrate similar trends up to the maximum load, followed by a reduction in stiffness.

The locations of the virtual DIC strain gauges and LVDT transducers are shown in Figure 7b. The principal tensile strain map at maximum load (Figure 7c) indicates a pronounced localisation of tensile strains along selected bed joints, forming several distinct localisation bands across the surface of the sample. The corresponding compressive strain map (Figure 7d) shows localised compressive concentrations associated with these zones. Compared to other reinforcement systems, the strain field exhibits stronger localisation, indicating a more concentrated damage pattern.

### 3.5. Summary of Mechanical Parameters

A quantitative summary of the experimental results is provided in Table 1. The reported values correspond to the mean results obtained from three specimens per configuration. The maximum load values for the reinforced specimens differ only slightly from those of the reference specimen, remaining within a narrow range. The calculated values of the secant elastic modulus, determined in accordance with EN 1052-1 [23] as the secant modulus at one third of the maximum load, vary depending on the type of reinforcement.

**Table 1:** Raw experimental results of the ultimate load for individual masonry specimens.

Specimen Type	Maximum Load Measured for Specimen			Statistical Descriptors		
	F1	F2	F3	Mean	Min–Max	Relative Range
	[kN]	[kN]	[kN]	[kN]	[kN]	[%]
Reference (unreinforced)	363	323	355	347	323–363	12
Steel truss reinforcement	348	375	364	362	348–375	7
Carbon fibre mesh reinforcement	335	314	361	337	314–361	14
Steel cord mesh reinforcement	305	372	330	336	305–372	20

The relative range is defined as (max min)/mean × 100%.

For reinforced samples, the elastic modulus values obtained from the DIC and LVDT measurements are generally consistent, with relative differences ranging from 4.4% to +11.5%. These differences reflect measurement methodology and local deformation effects rather than changes in global load-bearing capacity.

Because only three specimens were tested for each configuration, the experimental results are presented using descriptive statistics rather than formal statistical inference. To ensure transparency of the measurements, Table 1 reports the raw results obtained from the individual specimens, while Table 2 summarises the corresponding descriptive statistics together with the main mechanical parameters derived from the tests. In addition to mean values, median values and min–max ranges are provided to illustrate the variability of the experimental results without overinterpreting the limited dataset.

As shown in Table 1, the experimental scatter of the ultimate load values varied between the investigated configurations, with the lowest variability observed for steel truss reinforcement.

The elastic modulus was calculated according to EN 1052-1 [23] as the secant modulus determined at one third of the maximum load (F_max/3). The cross-sectional area was identical for all samples (A = 216,000 mm^2^) and was used solely for the calculation of compressive stress.

## 4. Discussion

The experimental results demonstrate that the influence of bed joint reinforcement in compression-governed masonry should be interpreted primarily in terms of damage evolution and strain localisation rather than global load-bearing capacity. Although the investigated reinforcement systems did not lead to a measurable increase in the ultimate compressive strength, clear differences were observed in the spatial development and redistribution of the strain fields, captured by full-field DIC measurements. For all specimens, the ultimate load values remained within a narrow range, with differences comparable to experimental uncertainty. This confirms that, under axial compression, bed joint reinforcement cannot be treated as a structural strengthening measure in terms of increasing compressive resistance. This observation is consistent with the distinction introduced in EN 845-3 between structural and nonstructural reinforcement and supports the classification of the investigated systems as nonstructural solutions intended primarily for crack control. Despite the similarity in the ultimate load levels, the full-field strain maps reveal substantial differences in damage mechanisms. In the unreinforced reference specimen, the location of tensile strain occurred early and developed in a highly concentrated manner along selected horizontal joints, leading to rapid crack coalescence and a brittle post-peak response. In contrast, the specimen reinforced with steel truss-bed joint reinforcement exhibited delayed strain localisation and a markedly more distributed tensile strain field. The reinforcement promoted the redistribution of the deformation over a larger surface area, reducing the intensity of the localised strain concentrations. This behaviour is reflected in the higher apparent secant stiffness obtained from global deformation measurements, which should be interpreted as a consequence of strain redistribution rather than an intrinsic increase in material stiffness. The remaining reinforcement systems, steel cords embedded in a fibreglass matrix and unidirectional carbon fibre mesh, showed limited effectiveness in modifying the damage process. Although local interactions between the reinforcement and the surrounding mortar influenced crack paths, the overall strain localisation patterns remained similar to those observed in the unreinforced specimen. The comparison of the investigated reinforcement systems indicates that steel truss reinforcement promotes the most effective redistribution of deformation, whereas the carbon fibre mesh and steel cord mesh systems exhibit more localised damage patterns. In these cases, the concentrations of tensile stress remained largely confined to individual joints of the bed, with limited redistribution to adjacent regions. This suggests that reinforcement stiffness, geometry, and anchorage conditions play a critical role in controlling strain localisation under compressive loading. The observed differences between the investigated reinforcement systems can be interpreted in terms of their stiffness, geometry, and anchorage conditions within the mortar joint. Reinforcement with higher stiffness and more stable geometry, such as the steel truss system, promotes redistribution of deformation and delays the formation of dominant localisation bands. In contrast, reinforcement systems based on flexible meshes or cords provide weaker mechanical engagement with the surrounding mortar and therefore have a more limited influence on strain redistribution. As a result, the localisation of tensile strains and crack development remains more concentrated in these configurations. The comparison between DIC-derived deformation measures and LVDT measurements further supports the reliability of the adopted monitoring approach. The observed differences between elastic modulus values obtained using both methods are attributable to local deformation effects and measurement scale rather than inconsistencies in the experimental data. Importantly, DIC measurements provide access to spatially resolved information that cannot be obtained from conventional point-based sensors, enabling direct observation of crack initiation, propagation, and interaction with reinforcement elements. From a material health monitoring perspective, the results highlight the limitations of force-based indicators, such as ultimate load or global stiffness, in assessing the effectiveness of non-structural reinforcement systems. Although these indicators do not capture differences in damage mechanisms, full-field strain measurements allow for a more nuanced evaluation of reinforcement performance based on damage evolution and crack morphology. In this context, DIC serves not only as a measurement technique, but also as a diagnostic tool capable of linking local damage processes with global structural response. It should be noted that the present study is based on a limited number of specimens and therefore does not aim to provide statistically generalisable conclusions. The observed behaviour is consistent with previous experimental studies on reinforced masonry, which indicate that reinforcement embedded in bed joints may have a limited influence on the ultimate compressive resistance, while significantly affecting the development of cracking and the redistribution of deformation. In this context, full-field DIC measurements provide valuable insight into the evolution of strain localisation patterns that cannot be captured using conventional point-based measurements. From a practical engineering perspective, the results obtained confirm that bed joint reinforcement in compression-governed masonry should be interpreted primarily as a crack control solution rather than a structural strengthening measure. Although the investigated systems do not significantly modify the ultimate compressive resistance, they can influence the evolution of cracking and the redistribution of deformation. Consequently, its effectiveness should be evaluated mainly in terms of serviceability performance and damage control rather than the ultimate load capacity. The present study is based on a limited number of samples (*n* = 3 per configuration) and therefore does not aim to provide statistically generalisable strength parameters. Its primary objective is to assess the diagnostic value of full-field DIC monitoring for identifying differences in damage evolution and strain localisation in masonry with different bed joint reinforcement systems. Consequently, the conclusions should be interpreted in a comparative sense and regarded as indicative of the investigated configurations.

## 5. Conclusions

This study shows that bed joint reinforcement in masonry subjected to axial compression does not lead to a measurable increase in ultimate load-bearing capacity and should therefore be classified as a non-structural solution. Full-field digital image correlation revealed significant differences in damage evolution and strain localisation that are not captured by global force-based indicators, with steel truss reinforcement promoting strain redistribution and delayed localisation. These results demonstrate the diagnostic value of full-field deformation monitoring for assessing the effectiveness of crack control measures in masonry structures.

## Figures and Tables

**Table 2:** Descriptive statistics and mechanical parameters of the investigated masonry configurations.

Specimen Type	Ultimate Load	Ultimate Stress	E Modulus		DIC and LVDT Results Difference
			DIC Result	LVDT Result	
	F_max_	σ_max_	E_DIC_	E_LVDT_	E_LVDT_/E_DIC_-1
	kN	MPa	MPa	MPa	%
Reference (unreinforced)	347	1.61	874	-	-
Steel truss reinforcement	362	1.67	2168	2417	11.5
Carbon fibre mesh reinforcement	337	1.56	1196	1144	−4.4
Steel cord mesh reinforcement	336	1.56	1254	1243	−0.9

## Data Availability

The original contributions presented in this study are included in the article. Further inquiries can be directed to the corresponding author.
